# Matters of Interest to You

**Published:** 1902-03

**Authors:** 


### MATTERS OF INTEREST TO YOU.

### VETERINARY INSPECTION AT THE BOSTON HORSESHOW.

THE Boston Horseshow is to be held under the auspices of the Boston Horseshow Company, April 14th to 19th, 1902. The prize-list is just out, and shows that the usual classes, so well known in previous Boston shows and in the great shows of New York, Philadelphia, Chicago, Kansas City, and San Francisco, will be well provided for. Entries close Saturday, March 22. The show will be held, as heretofore, in the spacious Mechanics' Building, where a ring has been laid down that is 80 feet wide and 140 feet long.

The regular prizes range from $50 to $150 first, $25 to $75 second, and a ribbon to $50 third.

The judges are well-known horsemen from the Eastern States and Canada. Among the Canadian judges is Dr. Andrew Smith, of Toronto.

The veterinarians are Drs. Lester H. Howard, W. S. Plaskett, and Thomas Blackwood. The rules relating to veterinary examinations are as follows :

"11. In case of horses being entered and not exhibited the entrance fees and charges for stalls and boxes will be forfeited unless a certificate under the hand of the exhibitor be lodged with the secretary on or before April 7, 1902, certifying that such non-exhibition is caused by death or contagious disease. Such certificate must be confirmed by a veterinary surgeon.

"24. No person shall enter the space inclosed or set apart for judging, or hold any communication with the judges or examining veterinary surgeons, without permission of the managers.

"27. The company will employ its own veterinary surgeons, who will be in attendance each day of the show, and will inspect such horses as deemed necessary by the judges. No charge for the services to be made to exhibitors.

"28. Horses will be measured in the shoes in which they come to the exhibition, and this measurement shall hold good during the show in all competitions. Special care should be taken that their measurement is correct, as in the case of a wrong entry the animal will be disqualified from taking a prize. Horses will be measured by the company's veterinary surgeons, from whose decision, upon the approval of the presiding judges, there will be no appeal.

"29. All horses shall be examined by the company's veterinary surgeons before entering the show ring. This rule also applies to measurement.

"30. All horses doctored or in any way artificially, improperly, or unfairly prepared or tampered with before coming to the show ring will be disqualified."

It is further provided, under the special rules on judging and instructions to judges, that

"3. No stallion kept for service, or brood-mare, shall be rejected as unsound unless suffering from one of the following hereditary diseases: Roaring, whistling, ringbone, spavin, navicular disease, or cataract.

"4. The results of all veterinary examinations will be regarded as confidential."

Under the conditions governing the hunter classes the following requirement is found:

"All horses to win prizes in the classes for qualified or green hunters must be pronounced hunting sound by the *veterinaries* [sic] of the company, and must carry a minimum weight of 140 pounds."

The soundness of the horses in harness is covered by this rule:

"All horses in these classes must be practically sound, have good manners, and all-around action. Horses doctored or in any way artificially, improperly, or unfairly prepared or tampered with before entering the show ring shall be disqualified."

Among the conditions for horses entered in the contest for a special prize of a silver cup, valued at $300, for the best horse suitable for a gig, it is required that "they must be sound and able to go a good pace." In the entry form there is a statement that must be signed by the exhibitor, wherein he agrees "to forfeit and pay to the company the sum of $100 as and for liquidated damages if the animal or any of the animals I may exhibit are suffering from any contagious or infectious disease."

---

### VALUE OF FARM ANIMALS.

THE following is the substance of a preliminary report from the Census Office:

The Census Bureau, in a report on domestic animals, fowls, and bees in the United States on June 1, 1900, announces that all the domestic animals in the United States have a probable value of at least $3,200,000,000. Of this amount the value of the animals on farms and ranges constitute over 93 per cent., and those not on farms 7 per cent. The total value of all domestic animals on farms and ranges was $2,981,054,115, against $2,208,767,513 in 1890. There was a gain in all parts of the country except in the North Atlantic States, where there was a decrease of horses, sheep, and swine, making a total decrease of 3 per cent. in value.

The live-stock on farms in the United States follows: Calves, 15,330,333; steers, 15,253,182; bulls, 1,315,566; heifers, 7,182,014; cows kept for milk, 17,139,674; cows and heifers not kept for milk, 11,583,253; colts, 1,313,476; horses, 16,952,664; mules, 3,271,697; asses and burros, 95,603; sheep, 61,605,811; swine, 62,876,108; goats, 1,871,252.

Since 1890 the number of sheep decreased everywhere except in the West. The increase there was more than sufficient to balance the loss elsewhere, and made the number of wool-bearing sheep for the nation 11 per cent. greater than in 1890.

The number of horses on farms increased except in the North Atlantic States. The gain over the census of 1890 was 20 per cent. if the colts are included with the totals of 1900, and 13 per cent. if excluded.

The mules on farms increased generally. The dairy cows on farms and ranges in 1900 numbered 4 per cent. more than the milch cows reported in 1890. Under the term milch cows were included in 1890 more cows than those reported in 1900 as "cows kept for milk" or "dairy cows." The real gain, therefore, is approximately 25 per cent.

Neat cattle other than dairy cows increased generally. Swine increased 9 per cent., although there was a slight decrease in the North Atlantic division. In the South Atlantic division the value of domestic animals increased 14 per cent., to $184,152,273 in 1900. In the North Central division the value increased 27 per cent., to $1,529,306,487. In the South Central the increase was 70 per cent., to $598,525,267, and in the Western 93 per cent., to $361,453,353.

Iowa leads all the States in the total value of its live-stock, while Texas ranks second. The former has an investment in live-stock of $271,844,034, and the latter has $236,227,434. Texas, however, has the greatest number of neat cattle, mules, and goats; but the average value of these and other animals being less than in Iowa, the pre-eminence in value rests with the latter-named State.

